# A single exercise session improves side-effects of chemotherapy in women with breast cancer: an observational study

**DOI:** 10.1186/s12885-019-6310-0

**Published:** 2019-11-08

**Authors:** Anna Johnsson, Ingrid Demmelmaier, Katarina Sjövall, Philippe Wagner, Håkan Olsson, Åsa B. Tornberg

**Affiliations:** 1 Department of Clinical Sciences, Lund, Cancer Epidemiology and Oncology, Lund University, Lund, Sweden; 2 Department of Oncology, Skane University Hospital, Lund, Sweden; 3 Department of Public Health and Caring Sciences, Uppsala University, Uppsala, Sweden; 4 Department of Clinical Sciences, Lund, Cancer Epidemiology, Lund University, Lund, Sweden; 5 Department of Health Sciences, Lund University, Lund, Sweden

**Keywords:** Acute exercise, Breast cancer, Chemotherapy, Side-effects

## Abstract

**Background:**

To measure changes in four common chemotherapy related side-effects (low energy, stress, nausea and pain) immediately after a single exercise session within the first week after treatment.

**Methods:**

Thirty-eight patients with chemotherapy-treated breast cancer, participating in a multi-centre randomised controlled study, the *Physical Training and Cancer study (Phys-Can)* were included in this sub-study. The Phys-Can intervention included endurance and resistance training. Before and after a single training session (endurance or resistance) within the first week of chemotherapy, energy and stress were measured with the Stress-Energy Questionnaire during Leisure Time, and nausea and pain were assessed using a Visual Analog Scale 0–10. Paired t-tests were performed to analyse the changes, and linear regression was used to analyse associations with potential predictors.

**Results:**

Thirty-eight participants performed 26 endurance training sessions and 31 resistance training sessions in the first week after chemotherapy. Energy and nausea improved significantly after endurance training, and energy, stress and nausea improved significantly after resistance training. Energy increased (*p* = 0.03 and 0.001) and nausea decreased (*p* = 0.006 and 0.034) immediately after a single session of endurance or resistance training, and stress decreased (*p* = 0.014) after resistance exercise.

**Conclusions:**

Both endurance and resistance training were followed by an immediate improvement of common chemotherapy-related side-effects in patients with breast cancer. Patients should be encouraged to exercise even if they suffer from fatigue or nausea during chemotherapy.

**Trial registration:**

NCT02473003, June 16, 2015.

## Background

Breast cancer is the most common type of cancer among women in Sweden with 7558 diagnoses in 2016 [1]**.** Incidence is increasing, but mortality is not, due to improved detection and treatment. A large proportion of patients receive adjuvant chemotherapy as treatment in addition to surgery. During chemotherapy side-effects such as fatigue, myalgia, nausea, diarrhoea and peripheral neuropathy are common [2].

Physical activity is health-enhancing and highly recommended even after a cancer diagnosis. There is strong evidence from meta-analyses that exercise leads to increased physical function and health-related quality of life, fewer or less severe side-effects and decreased fatigue and depression [3, 4]. Furthermore, epidemiological studies report that patients who exercise have a lower risk of recurrence and mortality compared with those who are not active [3, 5]. Exercise interventions have been well-tolerated and beneficial during and following cancer treatment, including during adjuvant chemotherapy for breast cancer [6].

All these positive effects are the results of adaptation to long-term exercise and occurs after a period of regular training. However, a single session of exercise also affects the individual during and immediately after. For example, several circulating components such as sex hormones, insulin, inflammatory markers, stress hormones and immune cells show short-lasting changes [7]. When comparing the immediate effects of a single bout of endurance training, between breast cancer survivors and healthy controls, Evans et al. found different responses between the groups. Breast cancer survivors had attenuated epinephrine, cortisol and lactate response and larger changes in glucose and free fatty acid concentrations, compared with healthy controls [8]. Thus, the immediate effects of training for patients with breast cancer seems to be different than for healthy controls according to previous research [7, 8].

The subjective experiences immediately after a single exercise session on energy and fatigue have been studied in non-cancer populations. Loy et al. performed a meta-analysis and found consistent evidence for the effects on energy, whereas effects on fatigue were inconclusive [9]. Hoffman & Hoffman studied the effect of a single session of exercise in the general population, and found improved vigour and decreased fatigue in exercisers but not in non-exercisers [10].

A few studies of immediate exercise effects have been performed in patients with cancer. One showed a reduction in cancer-related fatigue after exercise in 18 hospitalised patients with various types of cancer who underwent radiotherapy and/or chemotherapy [11]. Two studies investigated the immediate effect of a submaximal cycle ergometer test. In the first, state anxiety decreased in 34 breast cancer survivors after the exercise test, especially among those with high state anxiety pre-exercise [12]. In the other, exercise self-efficacy increased among 20 sedentary endometrial cancer survivors [13].

For many women with breast cancer chemotherapy might be the most stressful aspect, with up to 90% of patients reporting some level of distress. The distress is often related to the experience of side-effects [14, 15]. The frequency and severity of side-effects vary considerably. However fatigue, nausea and pain are common [16], and they could be a barrier to exercise [17, 18]. To the best of our knowledge, how one single exercise session during chemotherapy affects energy, stress, nausea and pain has not been studied in patients with breast cancer. Because exercise is an important part of cancer rehabilitation, more knowledge is desirable in order to best guide and support patients during chemotherapy. Thus, increased understanding of the immediate experience of a single exercise session is of central clinical importance.

The aim of this study was to measure changes in energy, stress, nausea and pain immediately after a single session of aerobic or endurance training within the first week after chemotherapy. Additional aims were to study if potential changes were [1] related to type of training (aerobic or endurance) and [2] previous exercise habit, and if the [3] changes remained 3 hrs after training was completed.

## Methods

### Study setting and participants

The current study was conducted as an observational sub-study within the *Physical Training and Cancer study (Phys-Can)*. Phys-Can was a randomised controlled multicenter trial that included patients with newly diagnosed breast, colo-rectal or prostate cancer (*n* = 600) at three University hospitals in Sweden. The design was 2 × 2 factorial with high or low-to-moderate intensity exercise with or without supplementary behaviour change techniques, and the intervention included both endurance and resistance training for 6 months. Endurance training was home-based after an introduction at the gym and followed up by coaches [19]. Participants used heart rate monitors to reach the right intensity and the exertion was monitored using Borg’s Rating of Perceived Exertion scale (RPE-scale) [20] after every endurance session. Resistance training was always supervised and was performed twice weekly at a public gym. To get familiar with the resistance training, all participants were introduced to the programme during the first 6 weeks. The resistance training was performed on machines and included seated leg press, chest press, leg extension, seated row, seated leg curl, and seated overhead press using dumbbells. The Omni-scale for self-reported perceived exertion [21] was used each resistance training sessions.

Participants for the present study were consecutively recruited from one site of the main study Phys-Can between October 2016 and April 2018. Eligibility requirements were breast cancer diagnosis, planned treatment with adjuvant or neoadjuvant chemotherapy and training according to the Phys-Can protocol. The eligible women received written information about the study and gave informed consent to participate.

### Measurements

Energy and stress were assessed by *The Stress – Energy Questionnaire during Leisure Time* (SEQ-LT). It is a modified version of the Stress-Energy Questionnaire (SEQ), which was developed to measure stress and energy at work [22]. The psychometric properties of the SEQ-LT were found to be satisfactory, in a population of workers in human service organisations, by Hadzibajramovic et al., 2015 [23]. The questionnaire is an adjective checklist with two dimensions, energy and stress. Each dimension is represented by three positively and three negatively oriented items. In the present study the question was “How do you feel right now?” The response alternatives for all items were: (0) not at all, [1] hardly, [2] somewhat, [3] fairly, [4] much and [5] very much. In the general population, the neutral value on the energy scale (neither passive nor active) is 2.7, and on the stress scale (neither stressed nor calm) is 2.4 [22].

Nausea and pain intensity were assessed using a *Visual Analogue Scale* (VAS). The VAS was graded from 0 to 10 cm, where 0 represented “no nausea” or “no pain” and 10 represented “worst nausea possible” or “worst pain possible”. The VAS is well established in clinical practice and in research for measuring nausea and pain intensity [24, 25]. Additionally, the VAS is considered valid and reliable for measuring nausea [26] and pain intensity [27] in populations with cancer.

Sociodemographic background data, performed exercise in the intervention and previous exercise habits measured by The Exercise Stage Assessment Instrument (ESAI), were collected from the Phys-Can study data [19]. Age, clinical tumour stage and type of chemotherapy were retrieved from Medical records*.*

### Procedures

**Fig. 1:**
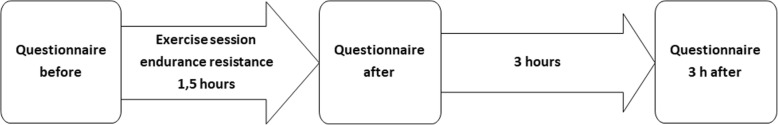
Questionnaire in relation to the training sessions

Women with breast cancer who participated in training at one site in the Phys-Can study were consecutively invited to the present sub-study. All participants performed both endurance and resistance training. Data was collected after the second treatment of chemotherapy. The instruction was to fill in the first questionnaire just before the training started (before), the second immediately after the training were completed (after) and the third 3 hrs after the training (3 h after), see Fig. 1. In the week before their second treatment of chemotherapy all the included participants received three identical questionnaires, to use at the first endurance training session in the week after treatment, together with instructions on how to fill them in. For the first resistance training session after the second treatment of chemotherapy the questionnaires were handed out to all participants and completed at the gym (before and after the session).

### Statistical analysis

Descriptive statistics were used for demographic and clinical characteristics of participants.

Scores for energy and stress were calculated as mean ratings of the six items in SEQ-LT after reversal of the items representing low energy and low stress. The response scale is 0–5 with higher ratings indicating higher level of both stress and energy [28]. Nausea and pain were reported on a Visual Analogue Scale (VAS) without numbers and entered as centimetres (0–10) from no pain/nausea (=0) to worst pain/nausea (=10) [29].

Differences in energy, stress and nausea, after a single training session were analysed using paired samples t-test and presented as mean changes, 95% Confidence Interval (CI) and Standard Deviation (SD). The significance level was set at 0.05. Sample size calculation was performed with G*Power, and a sample size of 34 individuals, performing one session of endurance training and one session of resistance training each, would provide > 80% statistical power to detect differences in energy assuming a two-tailed α = 0.05 and an effect size (ES) d = 0.5. We included in total 57 participants to take into account potential dropouts.

Comparisons were made between mean values before and immediately after training, and before and 3 hrs after training. Since both SEQ-LT and VAS are ordinal scales we also performed sensitivity analyzes using Wilcoxon signed ranks test. Participants with positive and negative change were presented as number of individuals and percent, and as a waterfall plot. Multiple linear regression was performed to analyse associations between the changes in energy or stress and the three following variables: previous exercise habit, training intensity and energy/stress-level before training. Non-significant variables were removed, and the model was re-fitted with only energy/stress levels before training. Residual distribution was examined graphically. In case they were deemed non-normal, bootstrap confidence intervals and *p*-values were generated as sensitivity analyses, to ensure that violations against residual distributional assumptions did not affect the study conclusions. Analyses were performed using IBM SPSS Statistics, version 25.

## Results

### Participants and baseline scores

**Fig. 2:**
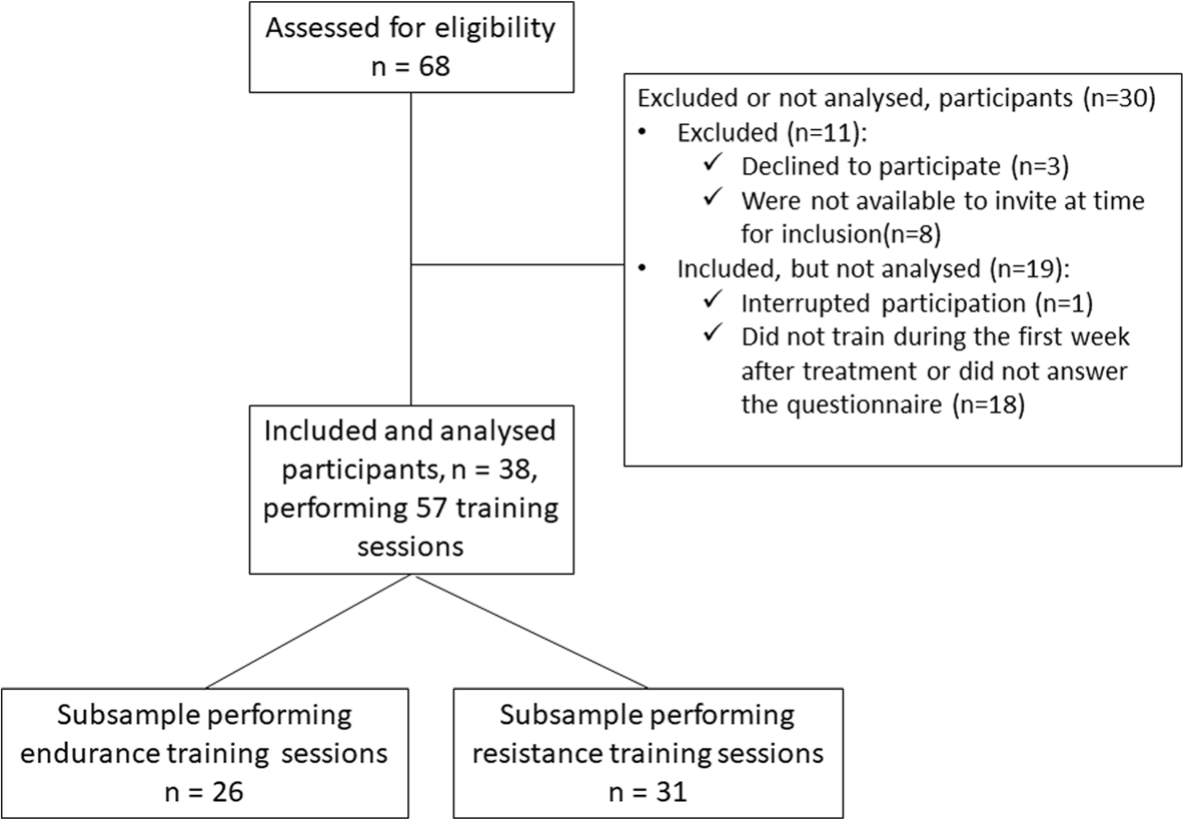
Study sample flowchart

Sociodemographic and clinical characteristics as well as baseline scores are presented in Table 1. Sixty-eight women were assessed for eligibility, 57 agreed to participate, among them 38 were included in the analyses. Nineteen of the 57 who agreed to participate did not train within the first week after treatment or did not answer the questionnaire, as described in Fig. 2.

**Table 1:** Demographic and clinical characteristics of participants

	All participants (*n* = 38)	Subsample performing an endurance training session (*n* = 26)	Subsample performing a resistance training session (*n* = 31)
Age (years)	53.6 ± 9.7	51.8 ± 8.6	53.5 ± 10.2
Habitual endurance exerciser ^a^			
Yes	18 (49%) ^b^	14 (54%)	15 (50%) ^c^
No	19 (51%)	12 (46%)	15 (50%)
Habitual resistance exerciser ^a^			
Yes	11 (29%)	9 (35%)	10 (32%)
No	27 (31%)	17 (65%)	21 (68%)
Education			
≤ 9 years	1 (3%)	1 (4%)	1 (3%)
9–12 years	8 (21%)	4 (15%)	6 (19%)
≥ 12 years	29 (76%)	21 (81%)	24 (77%)
Clinical stage			
I	16 (42%)	12 (46%)	13 (42%)
II	20 (53%)	12 (46%)	17 (55%)
III	2 (5%)	2 (8%)	1 (3%)
Treatment			
EC	35 (92%)	23 (88%)	29 (94%)
Docetaxel	2 (5%)	2 (8%)	1 (3%)
Kadcyla	1 (3%)	1 (4%)	1 (3%)
Before training			
Energy (score 0–5)	2.9 ± 1.0	2.8 ± 1.2	2.9 ± 1.1
Stress (score 0–5)	1.8 ± 0.8	1.8 ± 1.0	1.8 ± 0.9
Nausea (VAS 0–10)	1.6 ± 2.0	1.6 ± 2.3	1.9 ± 2.2
Pain (VAS 0–10)	0.6 ± 0.9	0.7 ± 1.1	0.4 ± 0.7

Continuous variables are presented as mean ± standard deviation (SD), and dichotomous or categorical variables are presented as number and percent. VAS visual analogue scale, EC Epirubicin and Cyclophosphamide

^a^ In maintenance phase according to ESAI (exercise Stage Assessments Instrument), i.e. regular endurance/resistance training at least six months before inclusion in the Phys-Can study

^b^ *n* = 37 due to missing information from one participant

^c^ *n* = 30 due to missing information from one participant

### Training sessions

#### Endurance training

Twenty-six participants performed a home-based endurance training session during the first week (mean number of days 2.4 ± 1.4) after chemotherapy. The perceived exertion on Borg’s RPE-scale [6, 7, 8, 9, 10, 11, 12, 13, 14, 15, 16, 17, 18, 19, 20] was median 13 [Q_1_ 11, Q_3_ 15] for participants in low-to-moderate intensity group (*n* = 15 and 2 missing values) and median 16 [Q_1_ 14, Q_3_ 16.5] for participants in the high intensity group (*n* = 5 and 4 missing values). The sessions lasted median 42 min, ranged from 15 to 120 min (*n* = 24 and 2 missing values).

#### Resistance training

Thirty-one participants performed a session of resistance training during the first week after chemotherapy. The mean number of days after chemotherapy was 2.9 ± 1.5. After each exercise the exertion was rated according to the Omni-scale (0–10). The median for all, based on each participant’s highest value, were 9 [Q_1_ 8, Q_3_ 9].

### Changes in energy, stress, nausea and pain

#### Endurance training

**Fig. 3:**
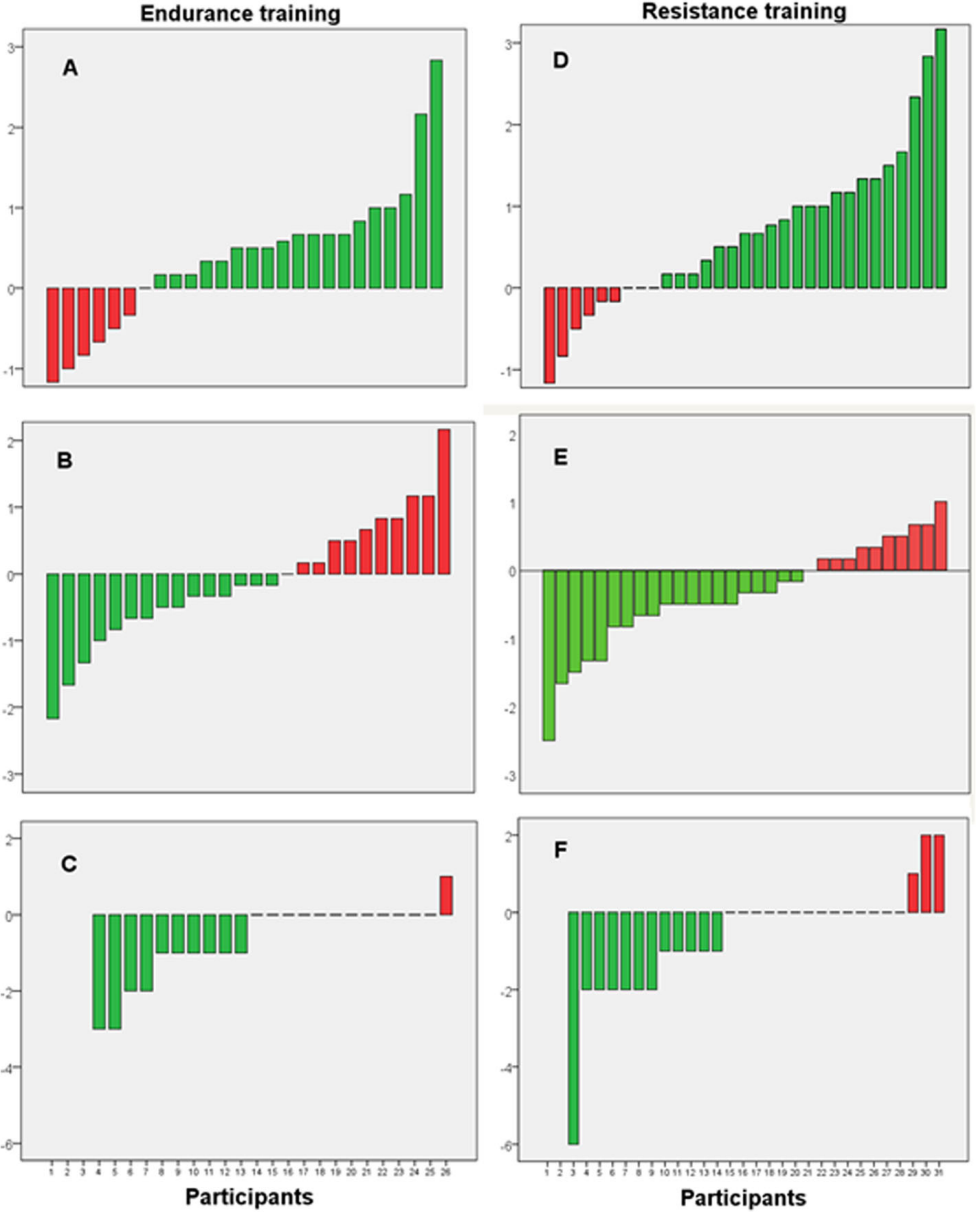
Number of participants reporting a positive (green) respective negative (red) changes in energy, stress and nausea from before to after a single session of endurance or resistance training. Endurance training: **a** Energy, **b** Stress, **c** Nausea. Resistance training: **d** Energy, **e** Stress, **f** Nausea

Before the training session the energy mean score was 2.8 and stress mean score was 1.9. Energy and nausea improved immediately after the training session, while stress did not (Table 2). The proportion of participants reporting an improvement in energy was 73% and in stress 58% (Fig. 3). Fourteen of the participants reported nausea before the training session and among them 10 (71%) improved, 2 (14%) were equal, 1 (7%) worsened immediately after the session (1 participant, 7%, had missing information after the session). Three hours after the training only stress was significantly improved compared to before the session. Lower energy before the training session was associated with larger increase in energy immediately after (B = − 0.53, 95% CI = − 0.75, − 0.31; *p* < 0.001). Higher stress score before training was associated with a larger decrease in stress after (B = − 0.65, 95% CI = − 0.94, − 0.35; p < 0.001). No significant associations were found between previous exercise habits and changes in energy (B = − 0.09; 95% CI = − 0.65, 0.47; *p* = 0.75) or stress (B = − 0.17; 95% CI -0.78, 0.43; *p* = 0.56), or between reported training intensity and changes in energy (B = 0.16; 95% CI = − 0.43, 0.76; *p* = 0.58) or stress (B = 0.14; 95% CI -0.55, 0.83; *p* = 0.68).

**Table 2:** Scores and changes in energy, stress and nausea after a single session of endurance training

	Before Mean ± SD *n* = 26	After Mean ± SD n = 26	3 h after Mean ± SD *n* = 25	Mean difference before-after (95% CI)	P^a^	Effect size (Cohen’s d)	Mean difference before-3 h after (95% CI)	P^a^	Effect size (Cohen’s d)
Energy (score 0–5)	2.8 ± 1.2	3.2 ± 0.8	2.8 ± 1.0	0.4 (0.0, 0.8)	0.030	0.45	0.1 (−0.4, 0.5)^b^	0.686	0.08
Stress (score 0–5)	1.8 ± 1.0	1.7 ± 0.8	1.4 ± 0.8	− 0.1 (− 0.5, 0.3)	0.585	0.11	− 0.5 (−1.0, 0.1)^b^	0.020	0.50
Nausea (VAS 0–10)	1.6 ± 2.3 ^b^	1.0 ± 1.9 ^c^	1.1 ± 1.9 ^c^	−0.7 (− 1.1, − 0.2)^c^	0.006	0.63	−0.5 (− 1.2, 0.2)^c^	0.164	0.30

SD Standard Deviation, CI Confidence Interval, VAS Visual Analogue Scale

^a^ Paired t-test

^b^ *n* = 25

^c^ *n* = 23

#### Resistance training

Energy, stress and nausea improved significantly immediately after the training session (Table 3). The proportion of participants reporting an improvement in energy was 71% and in stress 65% (Fig. 3). Fourteen of the participants reported nausea before training and among them 75% were improved, 6% were unchanged, and 19% reported worse nausea immediately after the session.

**Table 3:** Scores and changes in energy, stress and nausea after a single session of resistance training

	Before Mean ± SD *n* = 31	After Mean ± SD n = 31	3 h after Mean ± SD *n* = 29	Mean difference before - after (95% CI)	P^a^	Effect size (Cohen’s d)	Mean difference before - 3 h after (95% CI)	P^a^	Effect size (Cohen’s d)
Energy (scale 0–5)	2.9 ± 1.1	3.6 ± 0.8	3.0 ± 1.1	0.7 (0.3, 1.0)	0.001	0.69	0.1 (−0.3, 0.4)	0.754	0.06
Stress (scale 0–5)	1.8 ± 0.9	1.4 ± 0.7	1.2 ± 0.7	− 0. 4 (− 0.6, − 0.1)	0.014	0.47	−0.5 (− 0.8, − 0.2)	0.001	0.71
Nausea (VAS 0–10)	1.9 ± 2.2 ^b^	1.2 ± 2.0 ^b^	0.4 ± 1.1 ^c^	−0.6 (− 1.2, − 0.1)^b^	0.034	0.41	−1.3 (− 2.1, − 0.6)^c^	0.002	0.70

SD Standard Deviation, CI Confidence Interval, VAS Visual Analogue Scale

^a^ Paired t-test

^b^ *n* = 29

^c^ *n* = 26

At the measurement three hours after training the experience of stress and nausea was significantly improved, but not energy (Table 3). Lower energy before training session was associated with higher increase immediately after (B = − 0.64; 95% CI = − 0.89, − 0.38; *p* < 0.001). Higher stress level before training was associated with higher decrease (B = − 0.57; 95% CI = − 0.82, − 0.33; p < 0.001). No significant association were found between previous exercise habits and changes in energy (B = 0.03; 95% CI = − 0.56, 0.61; *p* = 0.93) or stress (B = 0.15; 95% CI = − 0.32, 0.62; *p* = 0.53).

#### Pain

Nine participants reported pain before training, which was located to lower back, stomach, chest and head. Immediately after the training session five out of these reported pains. Due to the small number of pain reports, we did not perform analyses on change.

## Discussion

This is, to the best of our knowledge, the first study describing changes in self-reported energy, stress and nausea immediately after a training session during chemotherapy. Despite the limited sample size, the results provide new and clinically relevant information. The results confirm the benefits of training during chemotherapy in terms of increased energy and decreased stress.

Energy was higher immediately after the training than it was before the session. We have not found any other study that measured energy immediately after a session of training in women with breast cancer during chemotherapy, but our results are similar to findings in non-cancer populations [9]. Most of the 16 studies in Loy et al.’s meta-analysis included healthy students, but four of them included participants with health concerns such as depressive disorder, persistent fatigue or bulimia nervosa. The most common intervention was endurance training on cycle ergometers, with an intensity from 40% of heart rate reserve to 75% of peak oxygen uptake (VO_2_ peak), and a duration from 10 to 40 min. Four of the studies evaluated resistance training (weight lifting), in college students [9]. The effect size in this meta-analysis is 0.47 on self-reported energy, which is similar to the effect size in our study with Cohen’s d = 0.45 for endurance, and d = 0.69 for resistance training. This effect size is clinically relevant, indicating that exercise during chemotherapy not only has beneficial long-term effects but also short-term. The changes are similar to that in studies of healthy individuals. The results are, therefore, important in clinical settings, when it comes to motivating patients to perform exercise even during chemotherapy.

Cancer-related fatigue (CRF) is defined by the National Comprehensive Cancer Network (NCCN) CRF as “a distressing, persistent, subjective sense of physical, emotional, and/or cognitive tiredness or exhaustion related to cancer or cancer treatment that is not proportional to recent activity and interferes with usual functioning” [30].. Lack of energy is one core aspect of fatigue, and our results show improved energy after a single training session. A recent study reported decreased CRF immediately after a single session of strength training or, walking training in patients with a various type of cancers who were hospitalised for radiation and/or chemotherapy. The study found decreased mean fatigue scores with a Cohen’s d effect size, of 0.74, and decreased or unchanged fatigue in 14 of the 18 patients [11]. It is well established that exercise is important in the management of CRF [4], and could be a method to enhance feelings of vigour and energy [31]. However, fatigue, lack of motivation or lack of confidence among cancer survivors could be reasons for non-participation in exercise programmes [32].

In the present study most of the participants reported higher or unchanged energy immediately after training, but there were some who reported less energy. Future studies should elucidate the reason for this variance. In general populations, mood-states such as vigour and fatigue immediately following exercise have been shown to be better in regular exercisers than in non-exercisers [10, 33]. It may be the same for patients with cancer, but we did not find any association between those who have a history of training and different response to a training session.

Self-reported stress decreased immediately after a resistance training session. After endurance training, stress was unchanged at group level, and some of the participants reported increased stress. The reasons for increased stress could be many. For example, it could be lack of time or fear of injury. Another possible explanation could be that most of the women in this study were on sick leave and therefore had a low stress score before training, or it could imply that SEQ-LT may assess another kind of stress than patients with cancer experience. The fact that the resistance training was performed in a group together with other women with the same diagnosis and same kind of treatment, and with a knowledgeable instructor, could be a reason for reductions in stress in those sessions [18], in contrast with individual endurance training.

Approximately half of the participants reported they had nausea before the training session, but only one of them reported worse nausea immediately after endurance training and three of them after resistance training. We did not have any information about medication for the nausea, which could be a potential confounder and could be a reason for the decreased nausea. However, both endurance and resistance training has been shown reduce chemotherapy-induced nausea as a long-term effect in patients with breast cancer [34]. Since we have not found any previous exercise studies reporting on immediate changes in nausea, our study contributes as one small piece of work to increase knowledge about exercise during chemotherapy.

The measurements three hour after the training, showed that the improvement in stress was maintained after both endurance and resistance training, and the improvement in nausea was maintained after resistance training, whereas the energy level was almost back to the before-training levels. Since we have no information about what the participants did in the time between the measurements, we cannot claim that the results at three hours after measurements were induced by the training.

## Limitations

As the design of this study is observational, we cannot claim the observed changes were caused by the exercise per se. The improvements we found could be due to social support, attention and expectation effects rather than effect of the training. However, the results are similar to previous studies and in line with known physiological and psychological responses to training. The comparison of endurance and resistance training could be unfair as they were performed in different contexts: resistance training was performed in a group setting with a coach knowledgeable about cancer, chemotherapy and its side-effects, and the endurance training was home-based. We did not control if the home-based endurance training was performed individually or together with others providing social support. In addition, we do not have information about what time of day exercise was performed. Training late at night or early in the morning could have affected the experience of both energy and stress.

The SEQ-LT questionnaire has not been used for a population with cancer before which could make it inappropriate to use in this setting. SEQ was developed to detect work-related stress and based on theories of changes in mood and emotions in response to environmental stimuli. However, it has since been modified and studied for use at leisure time in a population of workers in human service organisations, validated to measure non-work-related stress and energy [23]. Because we wanted a rather short questionnaire, that is easy to fill in at the gym for example, and we did not want to measure anxiety or fatigue (used before in populations with cancer), SEQ-LT was deemed to be relevant.

We could not perform analyses of pain due to the small number of participants (*n* = 9) who reported pain. Most of the participants, 88–94%, were treated with chemotherapy without side-effects such as pain. Whether granulocyte colony stimulating factors contributed to any pain was not possible to detect due to the size of missing data of medication. The one thing we can say, however, is that none of the initially pain-free participants reported pain after training.

The limited sample size prevented us from sub-group analyses. High-intensity endurance exercise has been reported to increase fatigue and low-intensity to increase vigour [35], and while this study had patients in these groups, the group size were small. Future studies should investigate the effect of the training intensity on the experience of energy immediately after the endurance training session.

## Conclusion

In the present study, we found positive changes in self-reported energy, stress and nausea immediately after a training session within the first week after chemotherapy. Our findings indicate that these common chemotherapy-related side-effects often improve after both endurance and resistance training. Patients should be informed about this as it could be a motivating factor for exercising. In future studies it will be important to investigate if the experience change during the course of chemotherapy.

## Data Availability

The datasets used and/or analyzed during the current study are available from the corresponding author on reasonable request.

## References

[1] The National Board of Health and Welfare. Cancer incidence in Sweden. 2018:2018 https://www.socialstyrelsen.se/Lists/Artikelkatalog/Attachments/20976/2018-6-10.pdf. .

[2] Nyrop KA, Deal AM, Shachar SS, Basch E, Reeve BB, Choi SK (2019). Patient-reported toxicities during chemotherapy regimens in current clinical practice for early breast Cancer. Oncologist..

[3] Cormie P, Zopf EM, Zhang X, Schmitz KH. The impact of exercise on Cancer mortality, recurrence, and treatment-related adverse effects. Epidemiol Rev. 2017:1–22. 10.1093/epirev/mxx007 28453622

[4] Fuller JT, Hartland MC, Maloney LT, Davison K (2018). Therapeutic effects of aerobic and resistance exercises for cancer survivors: a systematic review of meta-analyses of clinical trials. Br J Sports Med.

[5] Johnsson A, Broberg P, Kruger U, Johnsson A, Tornberg AB, Olsson H. Physical activity and survival following breast cancer. Eur J Cancer Care (Engl). 2019:e13037. 10.1111/ecc.13037 30895677

[6] Furmaniak AC, Menig M, Markes MH (2016). Exercise for women receiving adjuvant therapy for breast cancer. Cochrane Database Syst Rev.

[7] Dethlefsen C, Pedersen KS, Hojman P (2017). Every exercise bout matters: linking systemic exercise responses to breast cancer control. Breast Cancer Res Treat.

[8] Evans ES, Hackney AC, Pebole MM, McMurray RG, Muss HB, Deal AM (2016). Adrenal hormone and metabolic biomarker responses to 30 min of intermittent cycling exercise in breast Cancer survivors. Int J Sports Med.

[9] Loy BD, O'Connor PJ, Dishman RK (2013). The effect of a single bout of exercise on energy and fatigue states: a systematic review and meta-analysis. Fatigue: Biomedicine, Health & Behavior.

[10] Hoffman MD, Hoffman DR (2008). Exercisers achieve greater acute exercise-induced mood enhancement than nonexercisers. Arch Phys Med Rehabil.

[11] Matsugaki R, Akebi T, Shitama H, Wada F, Saeki S (2018). Immediate effects of exercise intervention on cancer-related fatigue. J Phys Ther Sci.

[12] Blanchard CM, Courneya KS, Laing D (2001). Effects of acute exercise on state anxiety in breast cancer survivors. Oncol Nurs Forum.

[13] Hughes D, Baum G, Jovanovic J, Carmack C, Greisinger A, Basen-Engquist K (2010). An acute exercise session increases self-efficacy in sedentary endometrial cancer survivors and controls. J Phys Act Health.

[14] Costanzo ES, Lutgendorf SK, Mattes ML, Trehan S, Robinson CB, Tewfik F (2007). Adjusting to life after treatment: distress and quality of life following treatment for breast cancer. Br J Cancer.

[15] Gibbons A, Groarke A (2018). Coping with chemotherapy for breast cancer: asking women what works. Eur J Oncol Nurs.

[16] Nyrop KA, Deal AM, Shachar SS, Basch E, Reeve BB, Choi SK, et al. Patient-Reported Toxicities During Chemotherapy Regimens in Current Clinical Practice for Early Breast Cancer. Oncologist. 2018. 10.1634/theoncologist.2018-0590 PMC6656489 30552158

[17] Henriksson A, Arving C, Johansson B, Igelstrom H, Nordin K. Perceived barriers to and facilitators of being physically active during adjuvant cancer treatment. Patient Educ Couns. 2016. 10.1016/j.pec.2016.01.019 26860549

[18] Lavallee JF, Abdin S, Faulkner J, Husted M. Barriers and facilitators to participating in physical activity for adults with breast cancer receiving adjuvant treatment: a qualitative meta-synthesis. Psycho-oncology. 2019. 10.1002/pon.4980 30657225

[19] Berntsen S, Aaronson NK, Buffart L, Borjeson S, Demmelmaier I, Hellbom M (2017). Design of a randomized controlled trial of physical training and cancer (Phys-can) - the impact of exercise intensity on cancer related fatigue, quality of life and disease outcome. BMC Cancer.

[20] Borg G (1970). Perceived exertion as an indicator of somatic stress. Scand J Rehabil Med.

[21] Robertson RJ, Goss FL, Rutkowski J, Lenz B, Dixon C, Timmer J (2003). Concurrent validation of the OMNI perceived exertion scale for resistance exercise. Med Sci Sports Exerc.

[22] Kjellberg A IA. Stress/Energi formuläret: Utveckling av en metod för skattning av sinnesstämning i arbetet [The Stress/Energy Questionnaire: Development of an Instrument for Measuring Mood at Work] Arbetsmiljöinstitutet1989.

[23] Hadzibajramovic E, Ahlborg G, Hakansson C, Lundgren-Nilsson A, Grimby-Ekman A (2015). Affective stress responses during leisure time: validity evaluation of a modified version of the stress-energy questionnaire. Scand J Public Health.

[24] Melzack R, Rosberger Z, Hollingsworth ML, Thirlwell M. New approaches to measuring nausea. CMAJ. 1985;133(8):755–758, 61. PMC1346462 4042058

[25] Price DD, McGrath PA, Rafii A, Buckingham B (1983). The validation of visual analogue scales as ratio scale measures for chronic and experimental pain. Pain.

[26] Borjeson S, Hursti TJ, Peterson C, Fredikson M, Furst CJ, Avall-Lundqvist E (1997). Similarities and differences in assessing nausea on a verbal category scale and a visual analogue scale. Cancer Nurs.

[27] Wilkie D, Lovejoy N, Dodd M, Tesler M (1990). Cancer pain intensity measurement: concurrent validity of three tools--finger dynamometer, pain intensity number scale, visual analogue scale. Hosp J.

[28] Kjellberg A, Wadman C. Subjektiv stress och dess samband med psykosociala arbetsförhållanden och hälsobesvär : En prövning av Stress-Energi-modellen. In Swedish. Subjective stress and its relation to psychosocial work conditions and health complaints. A test of the Stress-Energy model. Arbete och Hälsa: Arbetslivsinstitutet. 2002:34.

[29] Heller GZ, Manuguerra M, Chow R (2016). How to analyze the visual analogue scale: myths, truths and clinical relevance. Scand J Pain.

[30] Berger AM, Mooney K, Alvarez-Perez A, Breitbart WS, Carpenter KM, Cella D (2015). Cancer-related fatigue, version 2.2015. J Natl Compr Canc Netw.

[31] Puetz TW, Flowers SS, O'Connor PJ (2008). A randomized controlled trial of the effect of aerobic exercise training on feelings of energy and fatigue in sedentary young adults with persistent fatigue. Psychother Psychosom.

[32] Hardcastle SJ, Maxwell-Smith C, Kamarova S, Lamb S, Millar L, Cohen PA (2018). Factors influencing non-participation in an exercise program and attitudes towards physical activity amongst cancer survivors. Support Care Cancer.

[33] Hallgren MA, Moss ND, Gastin P (2010). Regular exercise participation mediates the affective response to acute bouts of vigorous exercise. J Sports Sci Med.

[34] van Waart H, Stuiver MM, van Harten WH, Geleijn E, Kieffer JM, Buffart LM, et al. Effect of low-intensity physical activity and moderate- to high-intensity physical exercise during adjuvant chemotherapy on physical fitness, fatigue, and chemotherapy completion rates: results of the PACES randomized clinical trial. J Clin Oncol. 2015. 10.1200/JCO.2014.59.1081 25918291

[35] Steptoe A, Cox S (1988). Acute effects of aerobic exercise on mood. Health Psychol.

